# Assessing the Effectiveness of Pirfenidone in Idiopathic Pulmonary Fibrosis: Long-Term, Real-World Data from European IPF Registry (eurIPFreg)

**DOI:** 10.3390/jcm9113763

**Published:** 2020-11-22

**Authors:** Ekaterina Krauss, Silke Tello, Jochen Wilhelm, Johanna Schmidt, Mark Stoehr, Werner Seeger, Ruth C. Dartsch, Bruno Crestani, Andreas Guenther

**Affiliations:** 1 European IPF Registry & Biobank (eurIPFreg/bank), 35392 Giessen, Germany; ekaterina.krauss@innere.med.uni-giessen.de (E.K.); silke.tello@innere.med.uni-giessen.de (S.T.); jochen.wilhelm@chemie.bio.uni-giessen.de (J.W.); johanna_weiser@hotmail.com (J.S.); mark.stoehr@innere.med.uni-giessen.de (M.S.); werner.seeger@innere.med.uni-giessen.de (W.S.); ruth.dartsch@innere.med.uni-giessen.de (R.C.D.); bruno.crestani@aphp.fr (B.C.); 2 Department of Medicine II, Universities of Giessen and Marburg Lung Center (UGMLC), Member of the German Center for Lung Research (DZL), 35392 Giessen, Germany; 3 Institute of Lung Health (ILH), 35392 Giessen, Germany; 4 Competence Center for Rare Pulmonary Diseases, Hopital Bichat, 75018 Paris, France; 5 Cardiopulmonary Institute, 35392 Giessen, Germany; 6 AGAPLESION Lung Clinic Waldhof-Elgershausen, 35753 Greifenstein, Germany

**Keywords:** idiopathic pulmonary fibrosis (IPF), European registry for idiopathic pulmonary fibrosis (eurIPFreg), interstitial lung diseases (ILD)

## Abstract

Background: Idiopathic pulmonary fibrosis (IPF) is a chronic progressive fibrotic pulmonary disease with rising incidence. In this study the effectiveness of pirfenidone, as measured by longitudinal change in individual slope of forced vital capacity (FVC) prior to and after initiating pirfenidone treatment, was evaluated in IPF patients recruited into the European registry for idiopathic pulmonary fibrosis (eurIPFreg). Secondary variables were the evaluation of the change in individual slope of diffusion capacity of the lungs for carbon monoxide (DLco), the Borg dyspnea scale, and six-minute walking distance (6MWD), as well as survival analyses. Results: Data of 122 eurIPFreg patients, who had at least two pulmonary function tests (PFTs) prior to or under treatment with pirfenidone, were analyzed by calculating slope-changes. The global analysis revealed an average slope change of +1.48 ± 0.28 (% per annum (p.a)) after start of treatment (*p* < 0.001), reflecting a reduction in annual FVC decline of approx. 50% under pirfenidone; it also showed a reduction in DLco, and increase in 6MWD (both *p* < 0.0001), as well as a flattening of the Borg dyspnea scale (*p* = 0.02). The median survival under treatment was 4.82 years. Patients with a more restrictive disease (FVC < 80% pred.), with a rapid progression (FVC decline >10% pred. p.a.), previous smokers and patients > 60 years of age seemed to profit more from pirfenidone treatment. Conclusions: We report the effectiveness of pirfenidone in a European “real world” IPF cohort with outcome data extending up to 9 years. Global analyses demonstrated a positive effect of pirfenidone on the decline of the lung function over time. Survival was dependent on Gender–Age–Physiology (GAP) score and age prior to therapy.

## 1. Introduction

Idiopathic pulmonary fibrosis (IPF) is a chronic, progressive, and usually fatal fibrotic pulmonary disease with rising incidence, being associated with an economic healthcare burden [1]. The natural history of this disease is characterized by a decline in lung function, worsening of symptoms and health-related quality of life, as well as early mortality, especially in familial cases [2,3].

As fibrosis advances and further impairs pulmonary physiology, affected patients experience a high burden of symptoms. Progressive dyspnea is the hallmark symptom of IPF and leads to significantly impaired exercise capacity. Patients also commonly experience non-productive cough and fatigue. Due to symptoms and the resultant impact on physical, social, and emotional well-being, patients with IPF suffer from decreased health-related quality of life (HRQL) [4,5]. A precise diagnosis is challenging, as it requires the skilled integration of clinical, radiographic and histopathologic findings that are rarely typical in appearance [6]. Moreover, IPF is often complicated by many respiratory comorbidities, such as pulmonary hypertension, obstructive sleep apnoea and lung cancer, which significantly worsen quality of life and life expectancy in these patients [7].

International consensus guidelines recommend that the diagnosis of IPF should be made on a multi-disciplinary level, calling for the exclusion of known causes of ILD and the presence of a usual interstitial pneumonia (UIP) pattern on high resolution computed tomography (HRCT) or lung biopsy [8]. In accordance with this advance of a practical approach to IPF diagnosis, the Fleischner Society published a position paper recommending the replacement of “possible UIP” terminology with “probable UIP”, describing the presence of reticulation and traction bronchiectasis in a basal and subpleural distribution in the absence of honeycombing, thus reducing the need for surgical biopsy [9].

The natural course of IPF is quite heterogeneous. IPF usually progresses gradually, but acute exacerbation of IPF may occur at any stage in the clinical course [10]. Greater impairment in forced vital capacity (FVC) or diffusion capacity of the lungs for carbon monoxide (DLco), and a greater extent of fibrotic changes on HRCT, are known mortality predictors in ILD patients [11]. Despite substantial advancements in the understanding of the pathogenesis of IPF having been achieved, the course, progression factors, biomarkers and the response to the treatment of an individual patient still cannot be reliably predicted [12,13,14,15]. 

Hence, it is necessary to identify reliable prognostic factors for the risk of lung function decline and the response to medical therapies in a non-selected patient cohort. Data from patient registries, collecting longitudinal data on IPF patients, have already helped to improve the understanding of the clinical characteristics, the impact that the disease has on their quality of life and survival, as well as current practices in diagnosis and management [16]. The data collected in IPF registries are complementary to data from the clinical trials because the cohorts evaluated in registries have a broader spectrum of disease severity and comorbidities, and can also be followed for a longer period of time [17].

Prior to the development of pirfenidone and nintedanib, there had been little therapeutic innovation in IPF treatment for several decades, and treatment options had provided very limited values in terms of either disease progression or physical performance. Pirfenidone (5-methyl-1-phenyl-2-[1H]-pyridone), was the first drug to be approved for use on IPF patients [18]. It is an anti-inflammatory and anti-fibrotic agent that down-regulates transforming growth factor beta (TGF-β) and tumor necrosis factor alpha (TNF-α), inhibits collagen synthesis and reduces fibroblast proliferation; the agent is licensed for the treatment of patients with mild-to moderate IPF in European Union and IPF in the United States [1,11]. 

Commercial release of pirfenidone in the European Union (EU) was granted based on the evidence from the two multinational pivotal phase III trials, PIPF-004 and PIPF-006 (collectively known as the CAPACITY trials) as well as a third supportive phase III study, conducted in Japanese patients [19,20]. The confirmatory phase III trial PIPF-016 (ASCEND) was conducted at the request of the USA Food and Drug Administration (FDA) with the results published in 2014 [21]. Further data of the long-term safety of pirfenidone were analyzed in the RECAP study [22]. 

## 2. Objectives of the Study

This is a retrospective secondary data use study to evaluate the effectiveness of pirfenidone in IPF patients. The primary endpoint was assessment of the treatment response, defined as change in annual rate of FVC decline after the start of pirfenidone treatment (t_0_) and required the existence of at least two PFTs prior to and two PFTs after the onset of therapy. 

Further primary objectives were to assess the effectiveness of pirfenidone, as measured by change in individual slope of FVC prior to and after initiating pirfenidone treatment in IPF patients (pts.) recruited into the eurIPFreg as well as in the following subgroups:

- pts. with limited functional impairment (>80% predicted FVC at t_0_);
- pts. with stable disease or slow disease progression before treatment (<10% decline of predicted FVC p.a.);
- pts. with progressive disease prior to treatment (>10% decline in FVC p.a.);
- pts. progressing after start of treatment (>10% decline in FVC p.a.);
- pts. with different smoking history (“yes” for active or previous smoker, “no” for never smoked pts.);
- pts. younger and older than 60 years;
- pts. with Gender-Age-Physiology (GAP) composite scores I, II and III;

Secondary variables were the evaluation of the effectiveness of pirfenidone treatment in the whole cohort, as measured by:

- change in individual slope prior to and after baseline (t_0_) of DLco (% predicted), Borg dyspnea scale (grades 0–10), and six-minute walking distance test (6MWD, meters);
- pts. surviving more than 24 months after the begin of treatment;
- survival analyses, presented by Kaplan–Meier curves;

## 3. Materials and Methods

We performed this study to evaluate the effectiveness of pirfenidone in IPF patients. The primary endpoint was defined as change in the annual rate of FVC decline after t_0_ in response to the treatment and required the existence of at least two lung function measurements prior to and after the onset of therapy. Secondary endpoints included change in annual rate of FVC under treatment in clinical subgroups and change in DLco, 6MWD, Borg dyspnea scale, as well as survival analyses.

The European IPF Registry (eurIPFreg) and the European IPF Biobank (eurIPFbank) were launched in November 2009 in the frame of the European IPF Network under the FP7 program to better explore the pathogenesis and natural course of IPF, also in order to facilitate translational research in biomaterials from IPF subjects [16]. Both, eurIPFreg and eurIPFbank have also been reviewed and received positive votes from institutional review boards in Germany (e.g., Ethics Committee of Justus-Liebig-University of Giessen; 111/08), France, Italy, Austria, Spain, the Czech Republic, Hungary and the UK. The research was conducted strictly according to the principles of the Declaration of Helsinki. The eurIPFreg and eurIPFbank are listed in ClinicalTrials.gov (NCT02951416).

On a local level, each patient’s IPF diagnosis was evaluated in a multidisciplinary discussion including at least chest physicians, pathologists and radiologists on the basis of the respective ATS/ERS/JRS/ALAT guidelines. The registry had no explicit exclusion criteria, thereby reducing selection bias. The clinical data were collected at the time of enrolment and in intervals 3 to 12 months thereafter (dictated by clinical routine). The patient questionnaire included the patient’s demographics, a detailed medical history making use of the WHO classification, complaints as well as report of co-morbidities [16]. After entry into the registry, each case was checked by a documentation officer for data quality and for internal plausibility of medical data and the diagnosis of IPF (e.g., hints for collagen/vascular diseases or hypersensitivity pneumonia in patient questionnaires).

Follow-up data were acquired in a similar way, making use again of patient and physician questionnaires including additional information on intermittent respiratory infections, working status, transplantation or any changes in the medication, also such events as death. Biological materials such as blood, bronchoalveolar lavage fluid (BALF) and tissue samples as well as exhaled breath condensates and electronic Nose (eNose) profiles were centrally recorded and managed in the centralized European IPF Biobank (eurIPFbank) located in Giessen, providing materials for further clinical research.

The study cohort was recruited between November 2009 and May 2018 from the following sites: Universities of Giessen and Marburg Lung Center, Germany, including the nearby Agaplesion Lung Clinic Waldhof-Elgershausen, as well as from Competence Center for Rare Pulmonary Diseases of Hopital Bichat in Paris, France.

## 4. Statistics

Data were collected from IPF patients with at least two subsequent records without antifibrotic treatment and by at least two records under pirfenidone treatment.

The data were analyzed in two different ways:

1. Individual analysis: slopes and slope-changes were taken as estimated from each individual patient.
2. Global analysis: the data from all patients were subtracted from the respective baseline values and then pooled. The pooled data was used to fit a global segmented regression model.

Baseline was defined as the value at the date of the first pirfenidone treatment (t_0_). Baseline values of all quantitative variables were interpolated using natural spline fits with two degrees of freedom through the available data. If only a single value was given, then this value was used as the baseline value. The baseline GAP score and Borg scale were taken to be the next available score value closest to the baseline date, but maximally one record earlier or later.

The changes in the key variable (change in FVC % predicted per year) were analyzed with segmented regressions by patient. The break-point was fixed at baseline t_0_. The baseline response value was taken to be the value at t_0_. The mean slope difference was tested with a t-test. Differences between mean slope differences in defined subgroups were evaluated within global segmented regression models (not accounting for patient-level data). Survival analyses were performed using Cox proportional hazards models. Effects estimated in the “global” analysis with *p* < 0.001 were considered statistically significant. In the other analyses, a *p* < 0.05 was regarded as significant.

All continuous and ordinal variables were shown as mean and standard deviation; categorical variables were separated into tables by number and percentage of the whole cohort (in %). All analyses were carried out with the statistics software R 3.4 (https://www.R-project.org). The baseline GAP score and Borg scale were taken to be the next available score value closest to the baseline date, but maximally one record earlier or later. Data of subsequent visits after a change in the treatment or after transplantation was disregarded.

Survival analyses were performed using Cox proportional hazards models. Time-series data were analyzed with segmented regression models fixing the break-points at t_0_ (first pirfenidone treatment). The values at t_0_ predicted by the segmented regression model were taken as the baseline response value for the respective patient.

## 5. Results

### 5.1. Descriptive Characteristic of the IPF Cohort

In the current analysis, change in FVC slopes pre/post treatment was assessed in 122 IPF subjects under pirfenidone treatment. The demographics and baseline lung function values at t_0_ are displayed in Table 1. The mean Vital Capacity (VC) at t_0_ was 64.5 ± 17.5% of the predicted value, the mean FVC at t_0_ was 63.0 ± 18.3% of the predicted value, indicating an already advanced state of the disease.

In our cohort, bronchoscopy was performed in 91 cases; the bronchoalveolar lavage (BAL) differential revealed elevated neutrophil (15.5 ± 17.9%) and eosinophil (4.5 ± 4.6%) counts in face of normal lymphocyte (10.2 ± 11.7%) and reduced macrophage counts (70.3 ± 21.0% of all cells).

### 5.2. Response to Treatment with Pirfenidone

#### 5.2.1. Individual FVC Slope Analysis

In the individual analysis, the slopes for each patient were calculated in an individual fashion on at least two PFTs prior to and after initiation of treatment, so that 122 statistically independent estimates for the slopes (before t_0_, after t_0_, and the difference of the slopes after and before t_0_) were obtained. Distribution of individual slope changes for patients with different numbers of PFTs (2–10) is shown in Supplementary Figure S1.

The distribution of number of patients by number of PFTs before and after t_0_ is presented in Supplementary Figure S2. As can be seen from these figures, the number of PFT’s was not significantly different between the period prior to and thereafter, and the slope change in response to treatment did not seem to be significantly different in dependency of number of PFT’s. Suggesting that an estimate of the individual slope change on the basis of at least two PFT’s was comparable to those in RCTs.

There was a relevant heterogeneity in the individual estimates, and the mean prior FVC slope change in response to treatment was not significantly different (*n* = 122; *p* = 0.32). The data regarding the treatment effect displayed a wide range between further FVC decline of 5-percentage points (%) p.a. and an improvement in FVC values up to 2% p.a. (Supplementary Figure S3). The follow-up in the study cohort was between 0.15 and 8.9 years with a median of 1.88 and an interquartile range from 0.91 to 2.97 years, as presented in Supplementary Figure S4.

The individual analysis was modified by taking at least three PFT measurements prior to and after pirfenidone treatment. The data were available for 106 patients. When doing so, there were also no significant changes between pre and post treatment initiation (*p* = 0.75).

#### 5.2.2. Global FVC Slope Analysis

**Figure 1:**
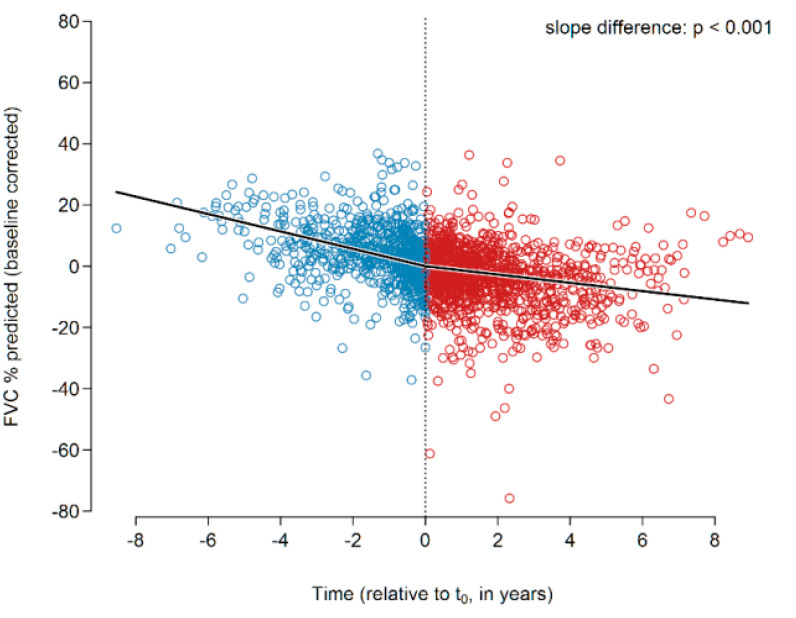
Changes in the FVC slope of the entire IPF cohort prior to (all blue circles) and after (all red circles) initiation of pirfenidone treatment. A significant change of the slope was encountered (*p* < 0.001). Abbreviations: FVC—forced vital capacity.

After setting baseline and performing individual FVC slope analysis individually as detailed above, all FVC data were corrected for the baseline values and analyzed together employing a global segmented regression, hereby disregarding the statistical dependencies of the patients’ recordings. In this analysis, the slopes and slope change were determined in a single segmented regression model using all data of all 122 patients and 2040 FVC values together (Figure 1). The slopes before and after t_0_ were −2.84 and −1.36% predicted p.a. (*p* < 0.001), respectively. The slope change at t_0_ was found to be +1.48 ± 0.28 (*p* < 0.001). Our results hence show a reduction in the annual FVC decline of roughly 50% under treatment with pirfenidone.

#### 5.2.3. Subgroup Analysis

For the subgroup analysis, the data were used as in the global analysis, but the segmented regression model included the interaction of the slope coefficients with the co-variable of interest, allowing testing of the null hypothesis that the slope differences in the subgroups are zero as well as the difference in slope-differences between the subgroups.

##### Patients with Limited Functional Impairment (>80% Predicted FVC at t_0_)

Data from patients with and without limited functional impairment showed a negative FVC slope before t_0_ (Supplementary Figure S5). However, the decline was less pronounced in patients with a preserved baseline lung function (FVC > 80% pred. at baseline; right panel, *n* = 22) as compared to patients with a relevant reduction in FVC at baseline (FVC < 80% pred. at baseline; left panel, *n* = 100). Both patient subgroups showed a reduction in the FVC decline in response to treatment, indicated by a positive value for the slope change. However, in patients with preserved baseline lung function (right part of right panel), this did not reach significance (*p* = 0.17), whereas in patients with a relevant reduction in baseline FVC, this change did reach statistical significance (*p* < 0.001). The slope changes in both subgroups were found to be statistically different (*p* < 0.001).

##### Response of Patients with Stable or Slow Progression versus Fast Progression before Treatment (>10% Decline of Predicted FVC p.a)

Data from patients with stable disease or slow disease progression, defined as a decline of FVC (in % pred) of less than 10% per year prior to treatment showed a slope of ~−4% before t_0_ (Supplementary Figure S6, right panel, left part, *n* = 98). In response to treatment, there was a significant (*p* < 0.001) reduction in the FVC decline in this patient sub-population, resulting in a positive value for the slope changes at t_0_ (Supplementary Figure S6, right panel, right part).

Data from patients with progressive disease prior to treatment, defined as a decline of the FVC (in % pred.) of more than 10% prior to treatment showed a negative FVC slope before t_0_ approximating 18% (Supplementary Figure S6, left panel, left part; *n* = 24). In response to treatment, there was a significant (*p* < 0.001) reduction in the FVC decline in this patient sub-population, resulting in an impressive slope change of around 16% at t_0_. The change in the slope-differences in response to treatment between both subgroups was not found to be statistically significant (*p* = 0.345).

##### Patients Progressing after Start of Treatment (>10% Decline in FVC p.a.)

Data from patients with progressive disease after initiation of treatment (t_0_), defined as an annual FVC decline of >10% pred., showed an FVC decline of ~3.2% pred. prior to treatment (Supplementary Figure S7, right panel, left part, *n* = 28), as compared to an FVC decline of ~2.4% pred. in patients who did not deteriorate after initiation of treatment (FVC slope <10% pred.; Supplementary Figure S7, left panel, left part, *n* = 94). Accordingly, patients with a deterioration after initiation of treatment showed a massive and highly significant (*p* < 0.001) worsening in the FVC slope (right panel, right part), as compared to the similarly significant (*p* < 0.001) slight improvement in the FVC decline in those patients stable upon initiation of pirfenidone treatment (left panel, right part). The change in the slope-differences in response to treatment between both subgroups was found to be statistically significant (*p* < 0.0001).

##### Never Smokers versus Patients with Smoking History

IPF patients in these two cohorts showed an FVC decline prior to treatment regardless of their smoking habits. However, current or former smokers with IPF (Supplementary Figure S8; right panel; *n* = 84) showed a somewhat less prominent decline in FVC prior to treatment (left part) as compared to never smoking IPF subjects (left part of left panel; *n* = 38). In current or former smokers, therapy with pirfenidone resulted in a significant (*p* < 0.001) reduction in FVC decline (right part of right panel). In never smoking IPF subjects the slope change was less prominent and it did not reach statistical significance (*p* = 0.056, right part of left panel). There was a significant difference between both groups with regard to the response to treatment (*p* < 0.001).

##### Patients Younger and Older than 60 Years

IPF subjects older than 60 years (Supplementary Figure S9, right panel, *n* = 93) showed a more prominent decline in the FVC prior to initiation of treatment (left part, right panel) as compared to patients younger than 60 years (left panel, left part). Response to treatment was statistically significant (*p* < 0.001) in case of the patients above 60 years of age (right panel; right part) and resulted in a reduction in the FVC decline. In patients under 60 years of age, there was also an attenuation of the FVC decline, which, however, it did not reach statistical significance (left panel, right part, *p* = 0.145). Response to treatment was statistically significant between the two groups (*p* = 0.022).

##### Patients with GAP Stages I, II and II

FVC decline prior to initiation of treatment was found to be dependent on GAP stages (Supplementary Figure S10; left parts). In response to treatment with pirfenidone, GAP I patients (*n* = 26) did not show a meaningful change in the FVC slopes, whereas GAP II patients (*n* = 51) showed a weakly significant reduction in FVC decline (*p* = 0.04), and GAP III patients (*n* = 26) showed a highly significant reduction in FVC decline (*p* < 0.001). There was a significant (*p* = 0.007) difference in the treatment responses between GAP I versus GAP II patients.

Secondary objectives were the evaluation of the effectiveness of pirfenidone treatment in the whole cohort, as measured by:

#### 5.2.4. Change in Individual Slope Prior to and after t_0_ of DLco (% pred.), Borg Scale (Dyspnea Grade 1–6), and 6MWD (m)

##### DLco

**Figure 2:**
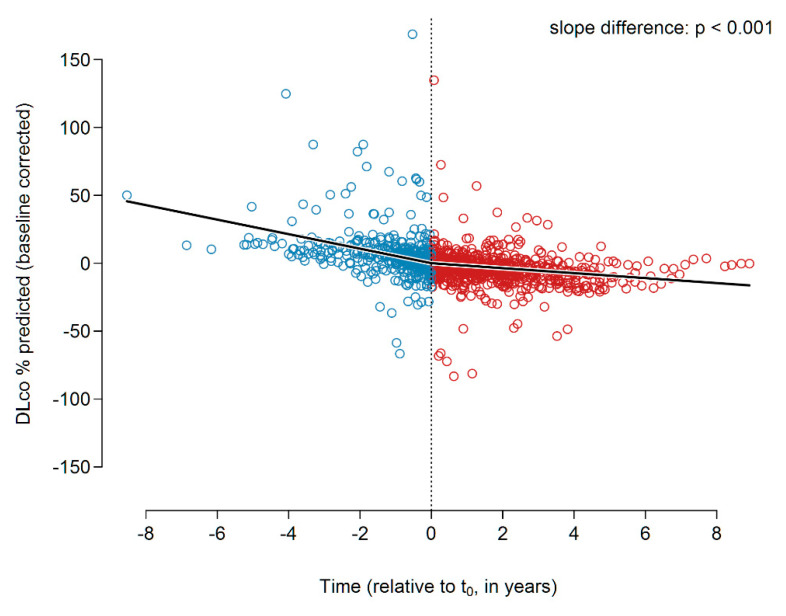
DLco slopes (% predicted) before and during pirfenidone treatment. Abbreviations: DLco—diffusion capacity of the lungs for carbon monoxide.

Although the individual slope analysis did not reveal any significant treatment effects, the global analysis revealed a significantly reduced DLco decline under pirfenidone. The slopes before and after t_0_ were −5.35 and −1.83% predicted p.a. (*p* < 0.001), respectively. The slope change at t_0_ was found to be +3.51 ± 0.63 (*p* < 0.001). Figure 2 presents the data.

##### Borg Dyspnea Scale

**Figure 3:**
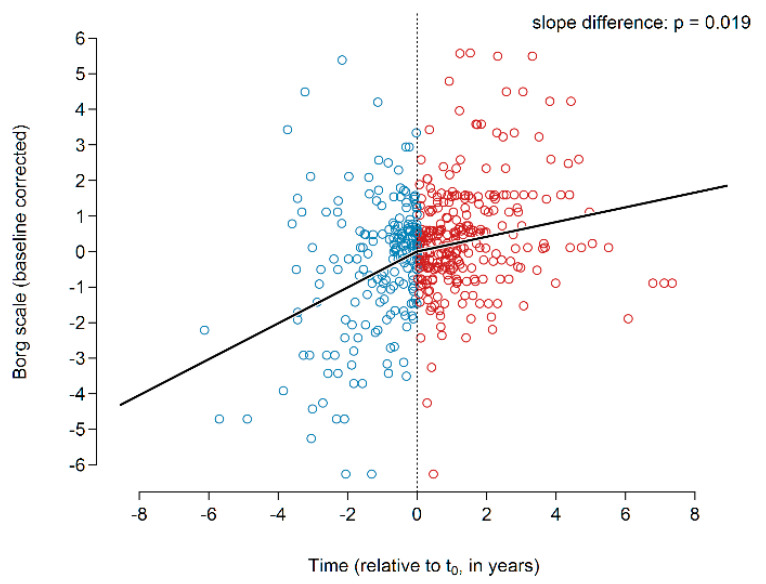
Borg dyspnea scale before and during pirfenidone treatment.

The global analysis showed an increasing Borg dyspnea grade prior to initiation of treatment and a significant attenuation of the dyspnea score in response to treatment with pirfenidone (*p* = 0.019; Figure 3, *n* = 43).

##### 6MWD

**Figure 4:**
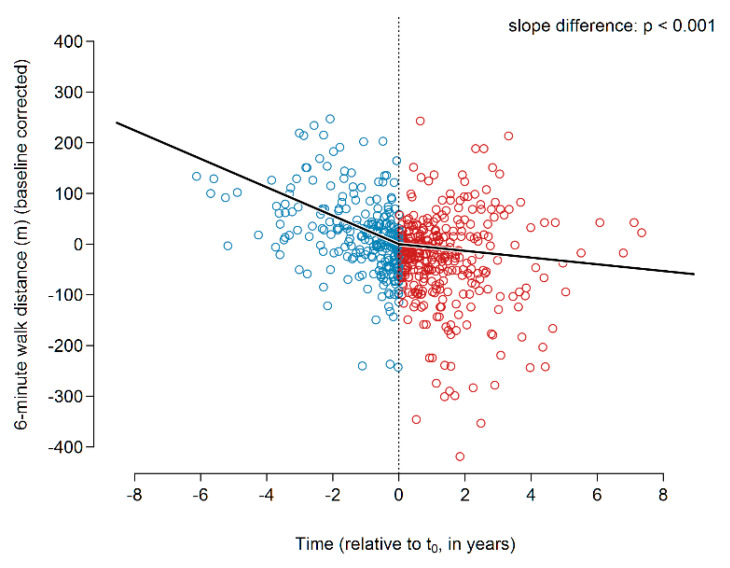
6MWD before and during pirfenidone treatment. Abbreviations: 6MWD—six-minute walking distance.

The global slope analysis showed a decline of 28 m per year in the 6MWD prior to the initiation of treatment (Figure 4). Upon initiation of pirfenidone treatment, the decline in 6MWD was found to be reduced, resulting in a slope of −6.6 m/year. This slope change was highly significant (*p* < 0.001).

Additionally, and in order to rule out a bias based on the drop out of deceased patients during the treatment period, we performed a subgroup analysis of the IPF cohort, who survived at least 24 months after beginning of pirfenidone therapy. In comparison to the whole IPF cohort, we saw similar pirfenidone effects on declines in FVC, DLco, 6MWD as well as improvement of the Borg scale. Supplementary Figures S11–S14 display the data.

#### 5.2.5. Survival Analysis, Presented by Kaplan–Meier Curves

##### Overall Survival

Survival time was analyzed starting from t_0_. The event (death) was only accepted if the patient died at the end of the valid treatment sequence. Dates of transplantation, changes in treatment or lost-of-follow ups were censored. The median survival under treatment was 4.82 years (95% confidence interval starts at 2.75 years, the upper limits could not be defined). The Kaplan–Meier curve is shown in Supplementary Figure S15.

##### Survival in Dependency of the FVC Decline Prior to Treatment

**Figure 5:**
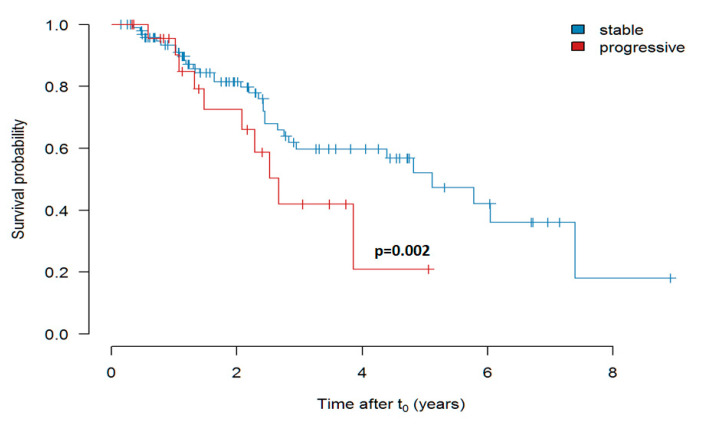
Survival of pirfenidone-treated patients with “stable” (FVC decline less than 10% p.a.) versus “progressive” (FVC decline more than 10% p.a.) disease prior to initiation of therapy (Kaplan-Meier curve shows estimate values). In this analysis the FVC slope was used as continuous predictor in a Cox proportional hazards (PH) model.

As shown in Figure 5, IPF patients with stable disease (FVC decline < 10% p.a.) prior to treatment showed a better survival after initiation of treatment as compared to patients with progressive disease (FVC decline > 10%). The effect of the disease progression before treatment with pirfenidone, as measured by FVC slope, was statistically significant (*p* = 0.002).

##### Survival in Dependency of Age Prior to Initiation of Treatment

As shown in Supplementary Figure S16, IPF patients under 60 years of age prior to treatment showed a much better survival after initiation of treatment as compared to patients over 60 years. The effect of age before treatment with pirfenidone, as measured by FVC slope, was statistically significant (*p* = 0.03).

##### Survival in Dependency of the FVC at the Timepoint of Treatment Initiation

**Figure 6:**
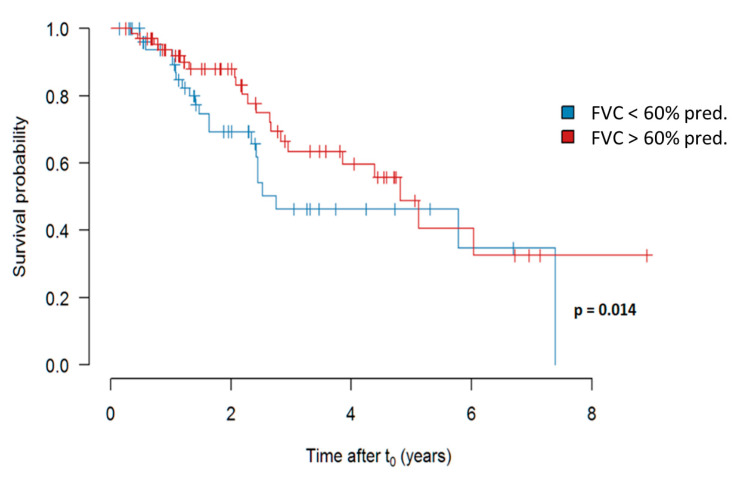
Survival in dependency of an FVC more (in red) versus less (in blue) than 60% predicted at the time of initiation of pirfenidone treatment. Abbreviations: FVC—forced vital capacity.

When analyzing the impact of FVC at the timepoint of treatment initiation, we did observe a weak, albeit significant (*p* = 0.014) impact of an FVC more than 60% versus less than 60% predicted on survival. The results are shown in Figure 6.

##### Survival Depending on GAP Stage at t_0_

**Figure 7:**
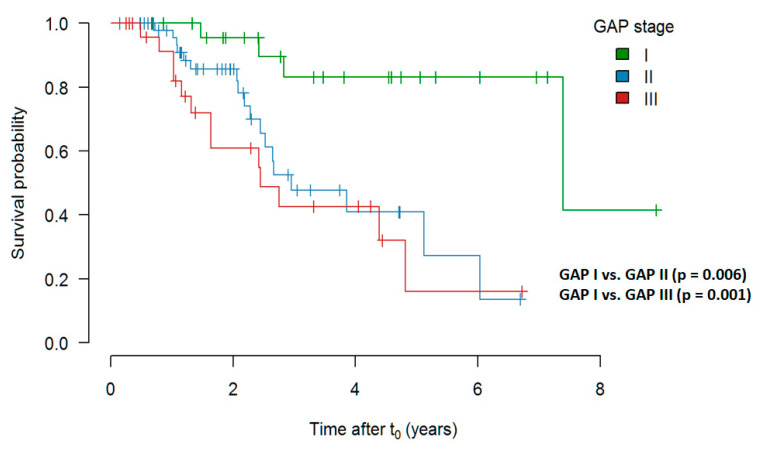
Survival depending on GAP stage at t_0_ (Kaplan–Meier curve shows estimate values). Abbreviations: GAP—Gender, Age, Physiology.

In comparison of pirfenidone treated patients with GAP stage I prior to initiation of treatment, stage II and III showed a significantly worse outcome. The data are presented in Figure 7.

##### Evaluation of a Risk Score Adjusted for FVC Decline, Age and GAP Prior to Initiation of Treatment

Based on a Cox regression analysis, we additionally performed a risk score analysis adjusted for the FVC decline and GAP stage prior to the initiation of pirfenidone treatment. The data are presented in Supplementary Figure S17 and show that, the higher the GAP score and the higher the FVC decline, the higher the risk of death in pirfenidone-treated patients.

**Figure 8:**
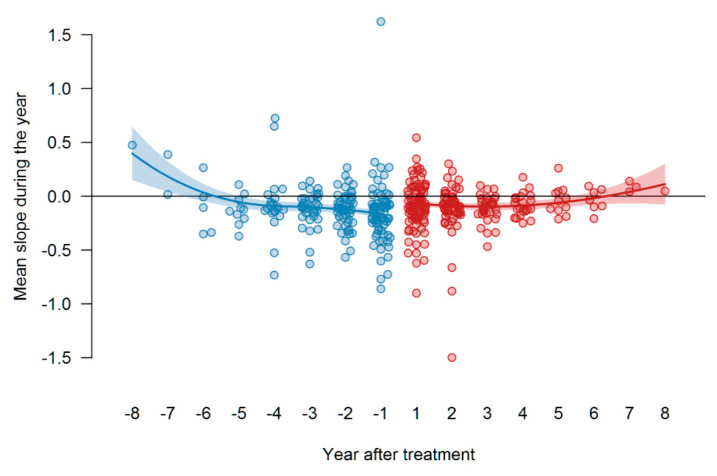
Therapy response to pirfenidone as measured by differences in the FVC slopes before and after t_0_. Figure 8 shows the values of FVC (in liters), blue dots represent values before t_0_, red dots—values after t_0_ (start of therapy). In addition, the points for the annual changes in the years before t_0_ are now also drawn. The year “0” is missing on the x-axis, the year “−1” is the change from year −1 to year 0 (=t_0_) (i.e., the period of the first year before t_0_), and the year “1” is the change from year 0 to year 1 (i.e., the period of the first year after t_0_).

To determine the mean changes per year in FVC, the FVC values per patient were fitted to a loess curve by a re-descending M estimator with Tukey’s bi-weight function and a span of 1 (year) and 12 iterations, showing a curve through the time course of the FVC values, predicted for t_0_ and at all years prior to and after treatment over the time range. The difference in these values between years was taken as estimates for the average yearly change in FVC. These values are shown for each patient as points. The means of these changes were determined separately for the years before and after treatment by the same procedure, but using the average yearly changes as data for fitting the loess curves. The two curves are shown together with their 95% confidence bands in Figure 8. These mean FVC values are shown as curves, along with their 95% confidence band, i.e., one can see that the changes become more negative shortly before t_0_ (the decline increases); the decline remains about constant and even increases again after a few years (so 5–6). The decline from the year before to the year after the start of therapy seems significant, but the *p*-value for the mean difference of these values (year −1 to year +1) was *p* = 0.12. Although the data spread wide, despite all the effort to extract dependent variances, treatment with pirfenidone seemed to be presumably equally effective throughout the whole treatment time.

The mean values and standard errors of the mean FVC slope decline per year (liters) are presented in Supplementary Table S1.

**Figure 9:**
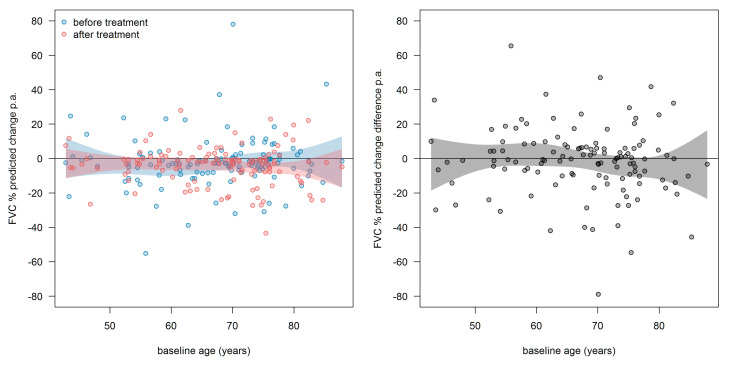
Distribution of the FVC slope and slope differences in dependency of age at t_0_. **Left**: slopes before (blue) and after (red) treatment. **Right**: slope-differences (after-before). Every point represents one patient. The curvy surfaces are the 95th confidence interval for mean values of age at t_0_. Abbreviations: FVC—forced vital capacity.

Additionally, we showed the distribution of the FVC slope and slope differences in dependency of age at t_0_ (Figure 9). The treatment response to pirfenidone did not depend on age at t_0_.

## 6. Discussion

This study provides a detailed analysis of pirfenidone elicited effects in a European “real world” IPF cohort. The global analyses of data pooled from repetitive measurements of 122 patients revealed a reduction in annual FVC decline of roughly 50% under treatment with pirfenidone, as well as a significant reduction in the DLco decline, and increase in 6MWD as well as a flattening of Borg dyspnea scale.

Data collected in the frame of randomized clinical trials (RCT) assessing efficacy of pirfenidone in IPF have provided important insights into the clinical course of IPF and the response to treatment. It should, however, be kept in mind that patients recruited into these studies represent a rigorously selected population and do therefore not necessarily reflect the characteristics of IPF subjects seen in clinical routine. Demographics and baseline measures of disease status of our study cohort are consistent with mild-to-moderate physiologic impairment and comparable to those in ASCEND and CAPACITY trials [22,23,24]. Eligibility criteria for the ASCEND study required a centrally confirmed diagnosis of IPF and a baseline DLco % pred. 30%, while the CAPACITY studies required an IPF diagnosis determined by site investigators and a baseline DLco % pred. 35% [25]. With the exception of an IPF diagnosis according to the consensus criteria, our “real-world data” had no explicit exclusion criteria, comprising a wide range of severity, progression, observation time (up to 9 years) and co-morbidities, thereby reducing selection bias and significantly complementing the data from RCTs. Despite these differences between our cohort and the cohorts studied in these RCTs, it is a striking observation that the effect size (around 50% reduction in FVC decline) seems to be pretty similar.

With regard to the analysis of change in FVC, patients with a more restrictive disease (FVC < 80% pred.), with a rapid progression (FVC decline > 10% pred. p.a.), previous smokers and patients > 60 years of age seemed to profit more from the pirfenidone treatment, in that the treatment elicited effects in these subpopulation appeared to be more profound. As an example, patients with progressive disease prior to treatment showed a negative FVC slope before t_0_ approximating 18% and, as response to treatment, a significant (*p* < 0.001) reduction in the FVC decline, resulting in an impressive slope change of around 16% at t_0_.

In our “real world” analysis of response to treatment with pirfenidone, GAP I patients did not show a meaningful decline prior to treatment and an only modest, non-significant change under treatment; GAP II patients showed a much more prominent FVC decline prior to, and a weakly significant reduction in FVC decline under treatment. Finally, GAP III patients showed the highest FVC decline prior, and a highly significant reduction in FVC decline under treatment. There was a highly significant difference in the treatment (FVC) responses between GAP I versus GAP III patients, whereas differences between GAP I and GAP II patients were not significant. Costabel et al. published data on pirfenidone treatment in 187 patients with more advanced disease, and 409 patients with less advanced disease; it was shown that longer-term pirfenidone treatment resulted in a similar rate of lung function decline and safety profile in patients with more advanced versus less advanced IPF [26].

Based on our individual slope analyses, the response to pirfenidone demonstrated a marked heterogeneity, hence rendering assessment of the individual treatment effect on disease progression quite challenging. The individually determined FVC slopes displayed a wide range between a further decline of 5% p.a. and an improvement of up to 2% p.a. After modifying the individual analysis by taking at least three PFT measurements prior to and after pirfenidone treatment (106 patients), there was still no visible significant treatment effect, although post treatment slopes were shifted to a lesser decline. This was reinforced by the finding that the intraindividually assessed slope changes in response to treatment were not substantially different if much more than two PFT’s were present for the slope analysis.

Survival time was analyzed starting from t_0_, where the event (death) was the end point (dates of transplantation, changes in treatment or lost-of-follow ups were censored). Our outcome analysis demonstrated a median survival under treatment of 4.82 years (95% confidence interval starts at 2.75 years, the upper limits could not be defined). In contrast to the substantial treatment effect on FVC seen in GAP III patients, patients at GAP stage III vs. GAP stage I (*p* = 0.001) showed a worse survival. When analyzing the impact of the FVC at the timepoint of treatment initiation, we did observe a weak, albeit significant impact of an FVC more than 60% versus less than 60% predicted on survival. Similarly, patients with a progressive disease (FVC decline > 10% per year) prior to therapy, although showing a striking increase in FVC, still showed a significantly higher mortality under treatment.

In a retrospective analysis of advanced IPF patients (with FVC < 50% pred.) and treatment with pirfenidone for at least 6 months, Tzouvelekis et al. showed a tendency towards a reduction in functional decline as compared to the 6-month period before treatment initiation, but failed to show any benefit after one year of treatment (*n* = 43). The authors presumed that efficacy of pirfenidone in IPF patients with severe lung function impairment may diminish after 6 months of treatment [27]. In a multicenter retrospective IPF study, Majewski et al. found that the median annual decline in FVC during the first year of pirfenidone therapy was −20 mL and during the second year was −120 mL (*n* = 307); this decline was even less than that noted in the ASCEND trial, in which mean decline from baseline in FVC was −235 mL in the pirfenidone group over 52 weeks [21,28]. Similarly, in another real-world study focusing on long-term use of pirfenidone in the large cohort (*n* = 502) of Japanese IPF patients, Bando et al. reported a mean decline of FVC of −30 mL and of −158 mL in the first and second year of therapy, respectively [29]. Moreover, a recent study of Vietri et al. reported similar findings of worsening of FVC decline rate after 12 months of pirfenidone treatment (*n* = 91) [7]. Taken together, these data may suggest that long-term efficacy of pirfenidone in IPF may differ according to the duration of treatment. Our data show that the response to pirfenidone is heterogeneous; one can see that the changes become more and more negative shortly before t_0_ (the FVC slightly declines as shown in Figure 8), but after that remain about constant and even increase again after a few years (5–6). In our cohort the median annual decline in FVC during the first year of pirfenidone therapy was −68 mL and during the second year was −90 mL (as presented in Supplementary Table S1).

Based on a Cox regression analysis, we additionally performed a risk score analysis adjusted for the FVC decline and GAP stage prior to the initiation of pirfenidone treatment. The data show that the higher the GAP score and the higher the FVC decline, the higher the risk of death under treatment. Fang et al. showed that IPF patients with severe baseline PFTs (DLco < 30%) were three times more likely to develop disease progression or acute exacerbation or death than the IPF subjects with DLco > 30% [18]. The median survival under treatment in the study by Vietri et al. was 1606 days (around 4.4 years) as compared to our research [7]. Ley et al. reported that treatment with pirfenidone when compared with placebo reduced the incidence of all-cause mortality at 1 year by 48%, improved progression-free survival and lowered the risk of respiratory-related hospitalizations [30]. IPF is strongly associated with age and older patients have a significantly worse prognosis with the disease [31]. Patients under 60 years of age prior to treatment showed a much better survival after t_0_ as compared to patients being over 60 years (*p* = 0.03).

Noble et al. analyzed pooled data from the multinational pirfenidone trials ASCEND and CAPACITY and found that treatment with pirfenidone for 1 year resulted in clinically meaningful reduction in disease progression in patients with IPF; analysis of outcomes at 1 year demonstrated that pirfenidone reduced the proportion of patients with a. ≥10% decline in FVC % pred. or death by 43.8% and increased the proportion of patients with no decline by 59.3% (95% CI 29.0–96.8) compared with placebo [19]. Antoniou et al. found that survival was higher in non-smokers than in former smokers or the combined group of former and active smokers [32]. Zurkova et al. evaluated the overall 2- and 5-year survival and lung function decline in 601 patients from the Czech EMPIRE registry who were diagnosed with IPF between 2012 and 2017. Of 601 patients, 63.7% of patients received pirfenidone treatment; this group had overall longer survival at 12, 24 and 60 months as compared to those treated with no antifibrotics [33]. More than half of the patients (55.9%) were still alive at the end of 5 years in the pirfenidone group as compared to 31.5% in the no antifibrotic group (*p* = 0.002).

Nathan et al. showed in the pooled analysis that at week 52, the relative risk of death for all four mortality outcomes was significantly lower in the pirfenidone group than in the placebo group in the pooled population (all-cause mortality *p* = 0.0107); treatment-emergent all-cause mortality (*p* = 0.0094); idiopathic-pulmonary-fibrosis-related mortality (*p* = 0.0029). Consistent with the pooled analysis, meta-analyses for all-cause mortality at week 52 also showed a clinically relevant and significant risk reduction in the pirfenidone group compared with the placebo group [34]. Albera et al. showed in post hoc analysis of pooled data from phase III RCTs that pirfenidone effectively reduced disease progression at 12 months as assessed by changes in different functional measures in both the baseline FVC < 80%/GAP stage II–III and FVC > 80%/GAP stage I subgroups [35]. Similarly, pirfenidone consistently reduced the categorical decline in 6MWD or death in both FVC < 80%/GAP stage II–III and FVC > 80%/GAP stage I subgroups, with comparable magnitude of treatment effect [35]. IPF is often complicated by many especially pulmonary comorbidities, such as pulmonary hypertension, obstructive sleep apnoea and lung cancer, that significantly worsen outcomes of these patients. Moreover, the patients who presented a later stage of the natural history of IPF would have lower survival chances due to the comorbidities and exacerbations.

## 7. Study Limitations

The interpretation of the real-world data has several limitations. First, as a databank, the information available for each patient is based on clinical practice instead on a trial protocol. Furthermore, in this study, we did not explicitly compare outcomes of pirfenidone versus so called “historic” controls, representing the natural course of the disease. Likewise, this research was not a part of a randomized clinical trial; therefore, no placebo group was evaluated.

## 8. Conclusions

In conclusion, we demonstrated a positive effect of pirfenidone-treatment on PFT decline, showing a reduction in annual FVC decline of approx. 50%, together with a reduction in the decline in DLco, 6MWD and flattening of the Borg dyspnea scale. Survival in the pirfenidone cohort was depending on GAP score and age prior to therapy. Patients with a more restrictive disease (FVC < 80% pred.), or rapid progression (FVC decline > 10% pred. p.a.) seemed to profit more from this therapy. Our results provide additional information as to the effectiveness of pirfenidone treatment on IPF disease progression and on treatment response in dependency of IPF-subgroups. Patients with a higher GAP score and higher age prior to treatment are more likely to experience a worse outcome despite treatment with pirfenidone. Our data highlights the important role of large real-world data registries to document and analyze changes in IPF management.

## Figures and Tables

**Table 1:** Clinical characteristics and baseline lung function in IPF patients at t_0_.

Parameters at t_0_	Pirfenidone Study Cohort
Patients in the analysis (*n*)	122
Male (%)	73.2
BMI (mean value ± SD (kg/m^2^))	28.3 ± 4.56
Age at t_0_ (mean value ± SD (years))	67.2 ± 10.3
Current smokers/previous smokers/never smoked (%)	4.1%/ 64.8%/31.1%
Pack years (mean value ± SD)	28.0 ± 21.0
GAP Stage I/II/III (% of the whole cohort)	21.1%/41.5%/21.1% (16.3% were missing values)
VC (% pred.; mean value ± SD)	64.5 ± 17.5
FVC (% pred.; mean value ± SD)	63.0 ± 18.3
DLco (% pred.; mean value ± SD)	42.4 ± 20.4
pO_2_ (mm Hg) at rest (mean value ± SD)	67.6 ± 72.4
pCO_2_ (mm Hg) at rest (mean value ± SD)	33.5 ± 44.9

**Abbreviations:** IPF—idiopathic pulmonary fibrosis, BMI—body mass index, DLco—diffusing capacity of the lung for carbon monoxide, FVC—Forced vital capacity, GAP composite score—Gender, Age, Physiology, pO_2_—partial pressure of oxygen, pCO_2_—partial pressure of carbon dioxide, VC—Vital capacity, % pred.—percent of predicted value.

## Supplementary Materials

The following are available online at https://www.mdpi.com/2077-0383/9/11/3763/s1, Figure S1: Distribution of individual slope changes in % FVC pred. p.a. in response to treatment and in dependency of PFT numbers (2–10) per patient, Figure S2: Correlation of number of patients to number of PFTs before and after pirfenidone therapy, Figure S3: Distribution of individual FVC slopes (in % pred.) prior to (in blue) and after (in red) initiation of treatment with pirfenidone (left panel), Figure S4: Distribution of follow-up times in the study cohort, Figure S5: FVC slopes in response to treatment in patients with preserved lung function (FVC >80% pred., right panel) versus those with more advanced disease (FVC <80% pred., left panel), Figure S6: FVC slopes (annual decline in % pred.) prior to initiation of treatment (left parts) and FVC slope changes after initiation of pirfenidone treatment (right parts) for patients with stable disease or slow disease progression before treatment (<10% p.a. in FVC decline, right panel) as compared to patients with progressive disease after start of treatment (>10% decline in FVC, left panel), Figure S7: FVC slopes (annual decline in % pred.) prior to initiation of treatment (left parts) and FVC slope changes after initiation of pirfenidone treatment (right parts) for patients with progressive disease after start of treatment (>10% decline in FVC, right panel) or for patients with rather stable disease after start of treatment (<10% decline in FVC, left panel), Figure S8: FVC slopes (annual decline in % pred.) prior to initiation of treatment (left parts) and FVC slope changes after initiation of pirfenidone treatment (right parts) for patients with a positive smoking history (current or former smoker; right panel) or in never smoking IPF subjects (left panel), Figure S9: FVC slopes (annual decline in % pred.) prior to initiation of treatment (left panels) and FVC slope changes after initiation of pirfenidone treatment (right panels) for patients over 60 years of age (right panel) or younger than 60 years (left panel), Figure S10: FVC slopes in % pred. prior to initiation of treatment (left parts) and FVC slope changes after initiation of pirfenidone treatment (right parts) for patients being in GAP I, II or III stage, Figure S11: FVC decline before and during pirfenidone treatment in patients surviving at least 24 months after onset of treatment, Figure S12: DLco decline before and during pirfenidone treatment in patients surviving at least 24 months after onset of treatment, Figure S13: Borg dyspnea scale before and during pirfenidone treatment in patients surviving at least 24 months after onset of treatment, Figure S14: 6MWD before and during pirfenidone treatment in patients surviving at least 24 months after onset of treatment, Figure S15: Kaplan-Meier curve of patients under pirfenidone treatment, Figure S16: Survival in dependency of the age at t_0_ (Kaplan-Meier curves), Figure S17: Scatter plot of the estimated risk (risk score with the highest number indicating the highest risk of death) depending on the FVC decline and GAP stage prior to initiation of treatment. Table S1: FVC decline per year under treatment with pirfenidone.

## Trial Registration

The eurIPFreg and eurIPFbank are listed in ClinicalTrials.gov (NCT02951416).

## Take Home Message

We demonstrated a positive effect of pirfenidone-treatment on PFT decline; showing a reduction in annual FVC decline of approx. 50%, together with a reduction in the decline in DLco, 6MWD and flattening of Borg dyspnea scale. Survival in the pirfenidone cohort was dependent on GAP score and age prior to therapy. Patients with a more restrictive disease (FVC < 80% pred.), or rapid progression (FVC decline > 10% pred. p.a.) seemed to profit more from this therapy.
