# Supplementary material for: Assessing the Effectiveness of Pirfenidone in Idiopathic Pulmonary Fibrosis: Long-Term, Real-World Data from European IPF Registry (eurIPFreg)
Source: J Clin Med. 2020 Nov 22;9(11):3763. doi: 10.3390/jcm9113763 (PMC7700641; doi:10.3390/jcm9113763)
Supplement: Supplementary file 1 [file jcm-09-03763-s001.pdf]

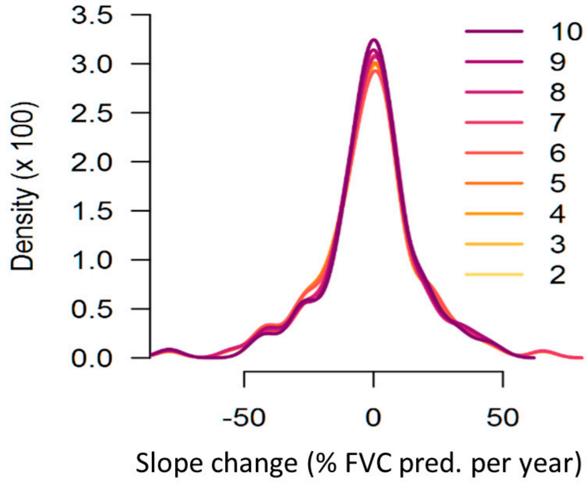

Suppl. Figure 1. Distribution of individual slope changes in % FVC pred. p.a. in response to treatment and in dependency of PFT numbers (2-10) per patient. The density curves show the distribution of individual slope changes. The colors refer to patients with different numbers of PFT. The interpretation is that (i) the distribution of slope change seems not to depend on the number of PFTs, and (ii) the distributions are very symmetric and are centered around 0. Abbreviations: FVC- forced vital capacity.

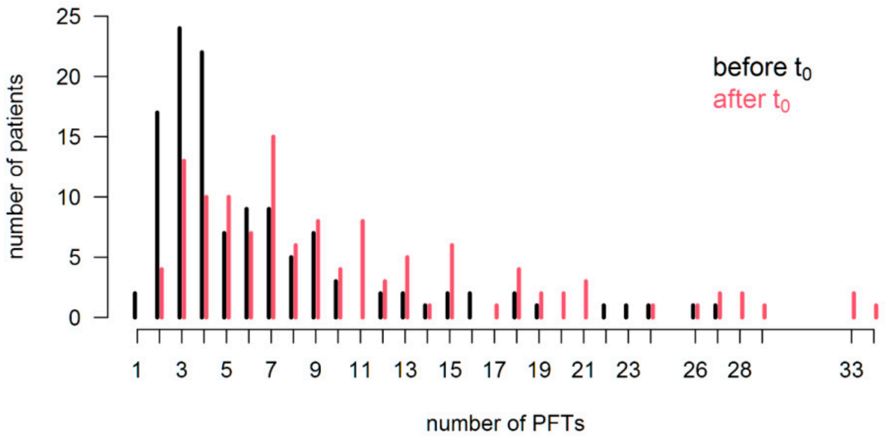

Suppl. Figure 2. Correlation of number of patients to number of PFTs before and after pirfenidone therapy. Presented are number of PFTs in correlation to number of patients. Abbreviations: PFT-pulmonary function tests.

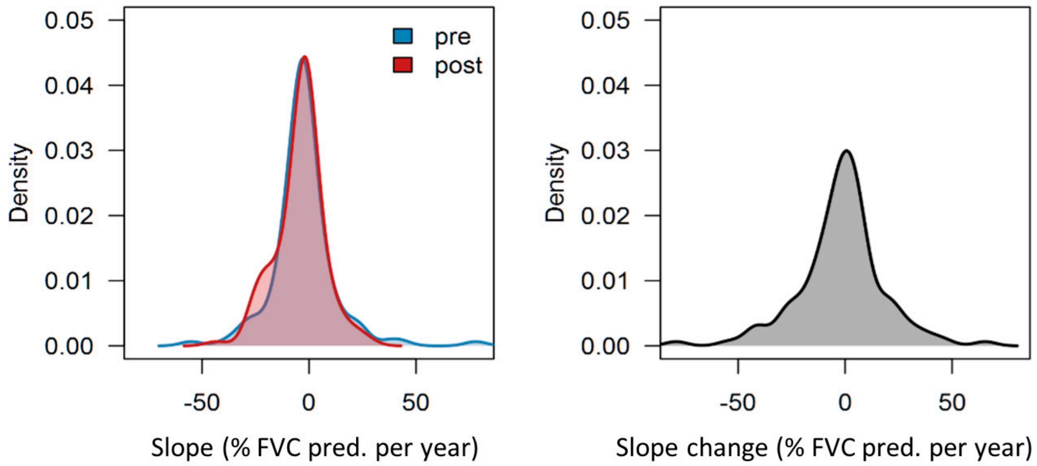

Suppl. Figure 3. Distribution of individual FVC slopes (in % pred.) prior to (in blue) and after (in red) initiation of treatment with pirfenidone (left panel). On the right panel, the distribution of the individual FVC slope change is depicted (n=122; p=0.32). Left panel shows the distribution of individual slopes (not slope changes) before (blue) and after (red) treatment. It shows that the distributions are quite symmetric and centered at zero. In this figure, the distribution curves have not been separated by the number of PFTs as in Suppl. Fig. 1. Hence, the estimation of each slope was done independent of the total number of PFTs. The right panel shows the slope change as shown in Suppl. Fig. 1, but, again, independent of the total number of PFTs available. Abbreviations: FVC- forced vital capacity.

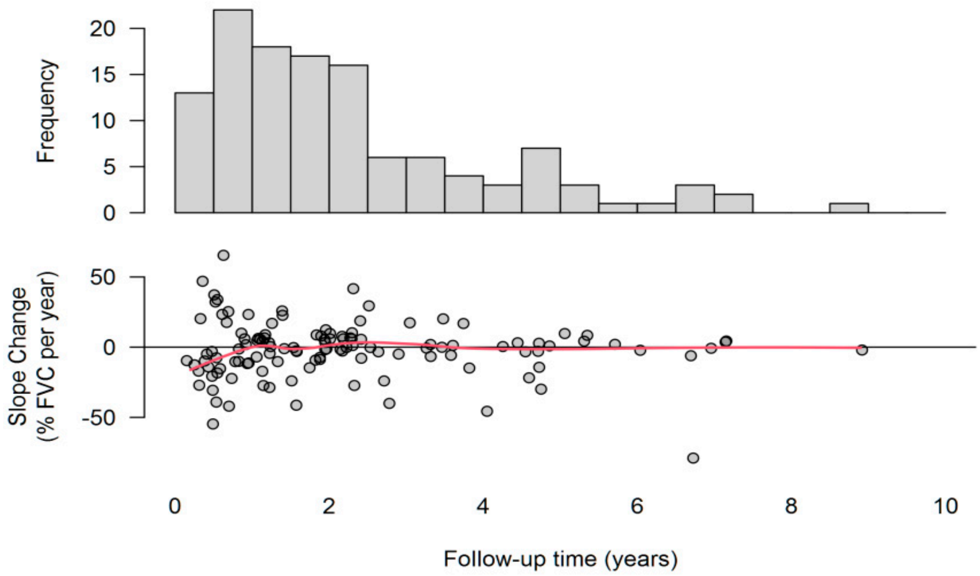

Suppl. Figure 4. Distribution of follow-up times in the study cohort. Follow-up was between 0.15 and 8.9 years with a median of 1.88 and an interquartile range from 0.91 to 2.97 years. The follow-up time ranged between 0.15 and 8.9 years, with a median of 1.88 and an interquartile range from 0.91 to 2.97 years. The lower panel shows the relation of follow-up time and slope change (%FVC per year). The curved red line is based on the re-descending M estimator with Tukey's bi-weight function and span 0.5. It illustrates the average slope change in dependency of follow-up time. There is no evident relationship between follow-up time and slope change. Abbreviations: FVC- forced vital capacity.

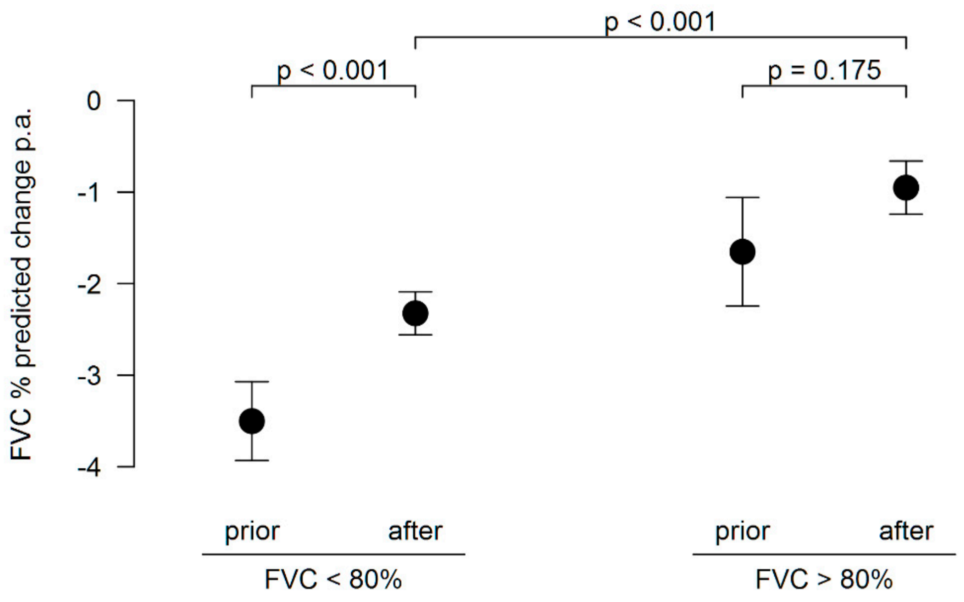

Suppl. Figure 5. FVC slopes in response to treatment in patients with preserved lung function (FVC >80% pred., right panel) versus those with more advanced disease (FVC <80% pred., left panel). The left parts of both panels indicate the FVC slope prior, the right parts show FVC slope after initiation of treatment. Left panel n=100, right panel n=22. Data represent mean values and standard errors. Abbreviations: FVC- forced vital capacity.

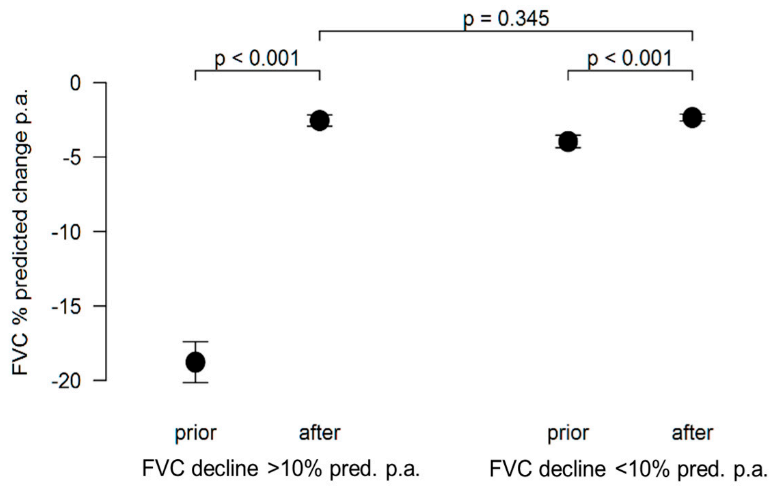

Suppl. Figure 6. FVC slopes (annual decline in % pred.) prior to initiation of treatment (left parts) and FVC slope changes after initiation of pirfenidone treatment (right parts) for patients with stable disease or slow disease progression before treatment (<10% p.a. in FVC decline, right panel) as compared to patients with progressive disease after start of treatment (>10% decline in FVC, left panel). Data represent mean values and standard errors. Abbreviations: FVC- forced vital capacity.

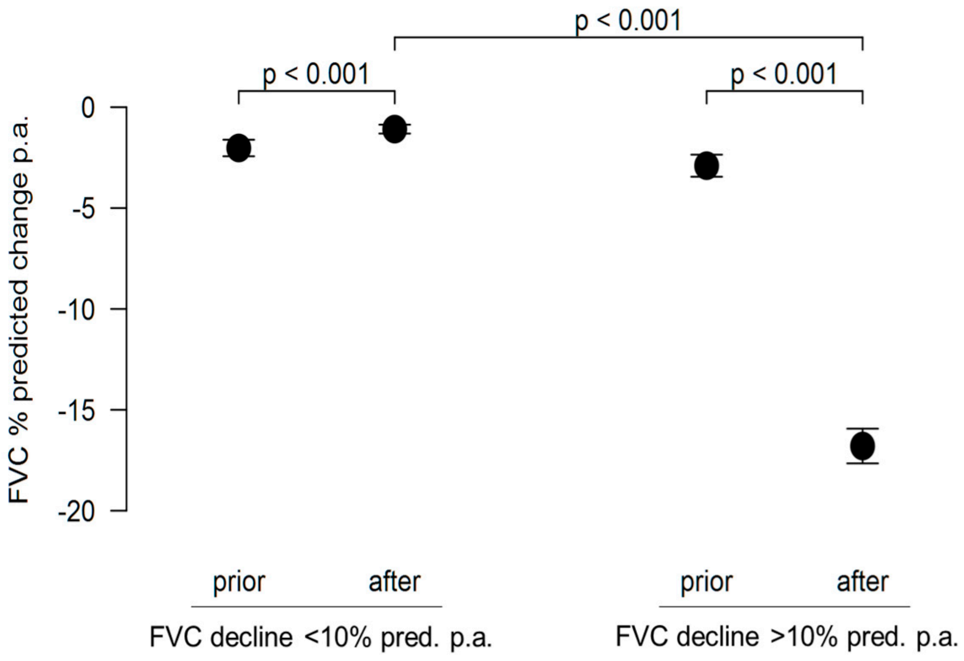

Suppl. Figure 7. FVC slopes (annual decline in % pred.) prior to initiation of treatment (left parts) and FVC slope changes after initiation of pirfenidone treatment (right parts) for patients with progressive disease after start of treatment (>10% decline in FVC, right panel) or for patients with rather stable disease after start of treatment (< 10% decline in FVC, left panel). Data represent mean values and standard errors. Abbreviations: FVC- forced vital capacity.

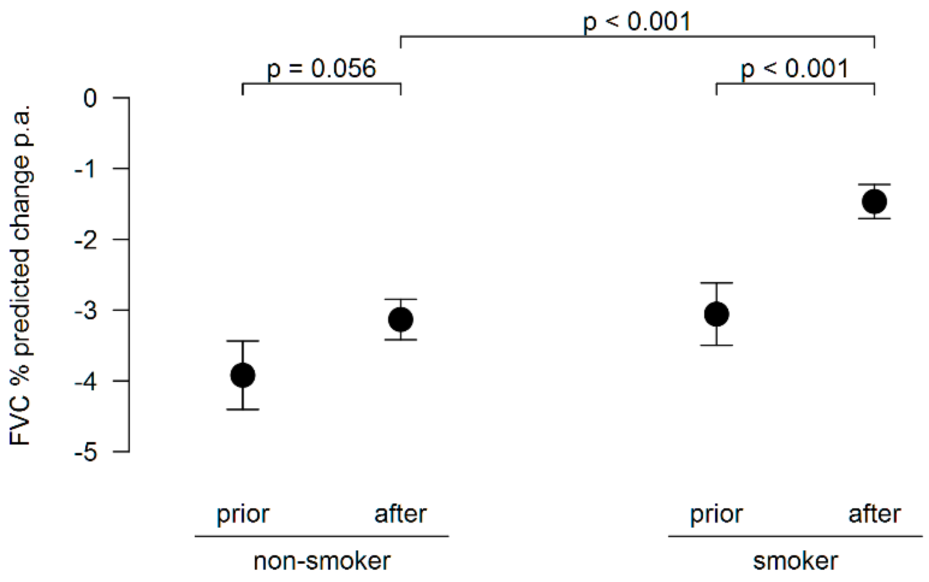

Suppl. Figure 8. FVC slopes (annual decline in % pred.) prior to initiation of treatment (left parts) and FVC slope changes after initiation of pirfenidone treatment (right parts) for patients with a positive smoking history (current or former smoker; right panel) or in never smoking IPF subjects (left panel). Data represent mean values and standard errors. Abbreviations: FVC- forced vital capacity.

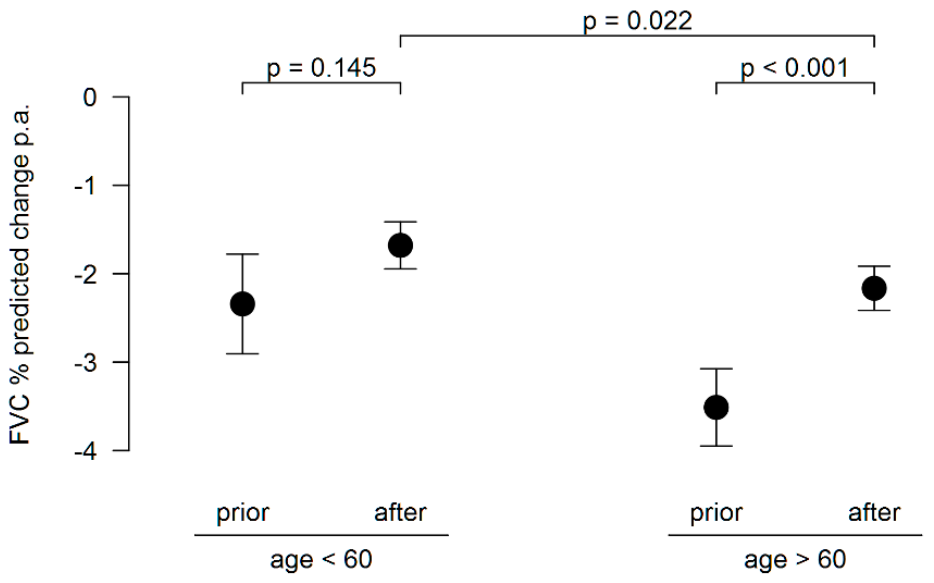

Suppl. Figure 9. FVC slopes (annual decline in % pred.) prior to initiation of treatment (left panels) and FVC slope changes after initiation of pirfenidone treatment (right panels) for patients over 60 years of age (right panel) or younger than 60 years (left panel). Data represent mean values and standard errors. Abbreviations: FVC- forced vital capacity.

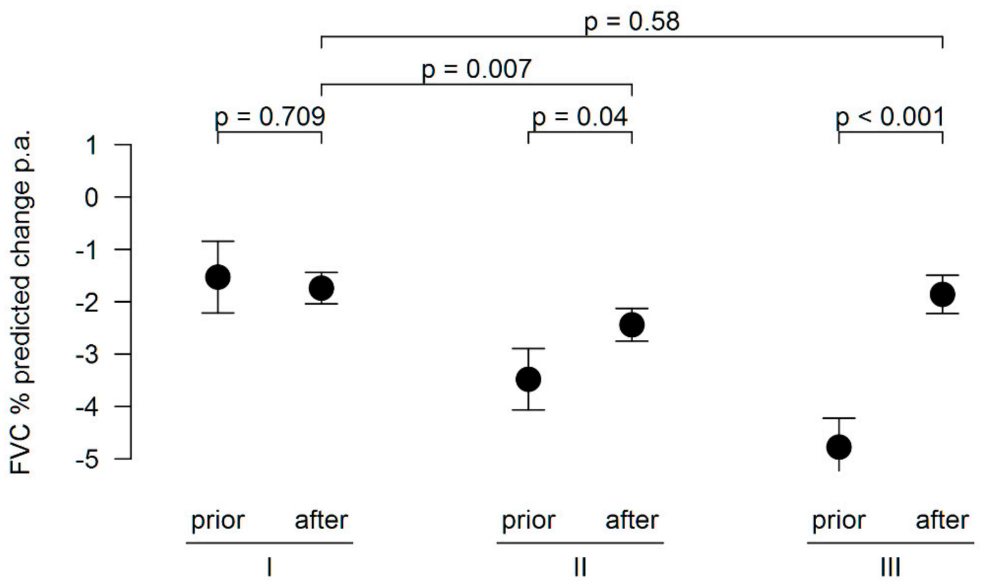

Suppl. Figure 10. FVC slopes in % pred. prior to initiation of treatment (left parts) and FVC slope changes after initiation of pirfenidone treatment (right parts) for patients being in GAP I, II or III stage. Data represent mean values and standard errors. Abbreviations: FVC- forced vital capacity, GAP- Gender, Age, Physiology.

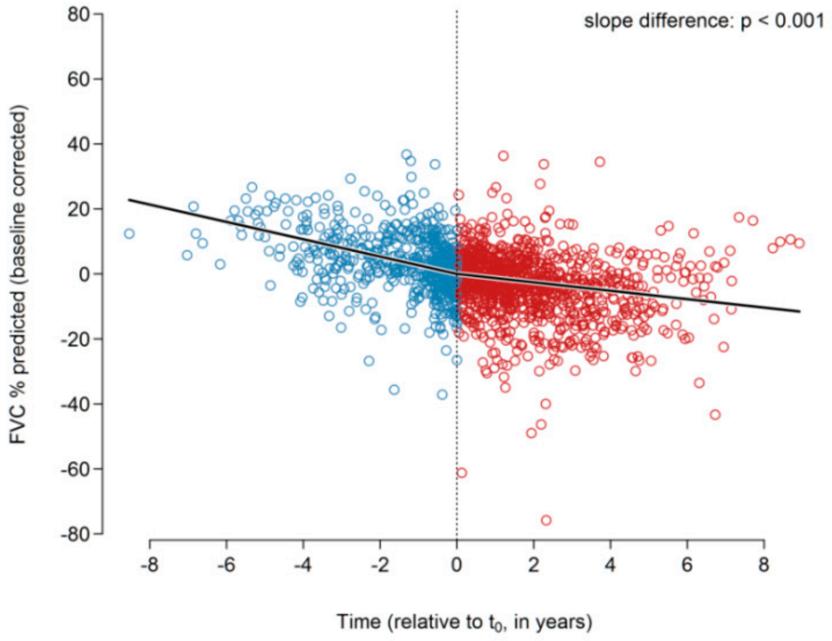

Suppl. Fig. 11. FVC decline before and during pirfenidone treatment in patients surviving at least 24 months after onset of treatment. Abbreviations: FVC- forced vital capacity.

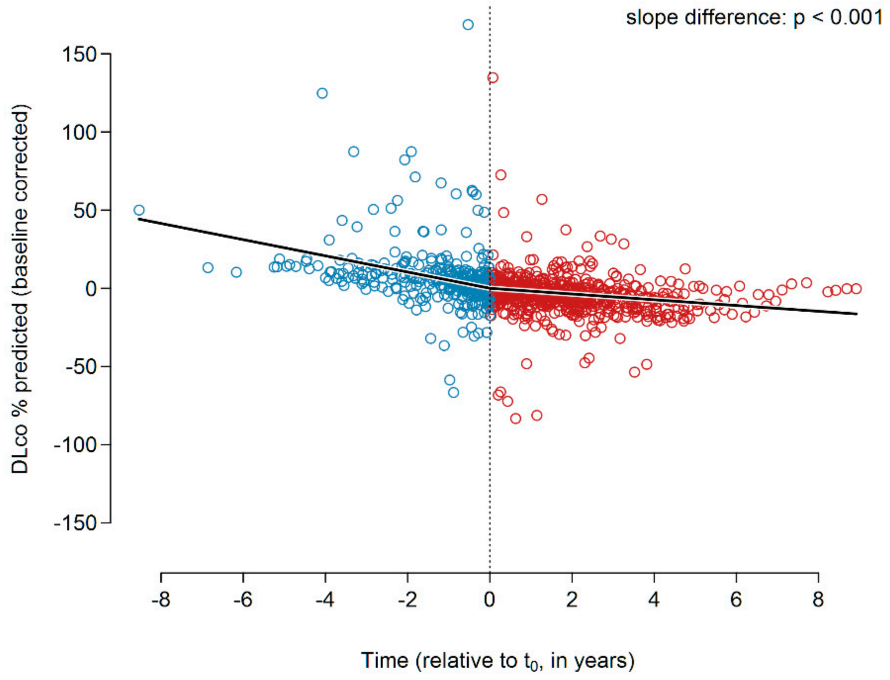

Suppl. Fig. 12. DLco decline before and during pirfenidone treatment in patients surviving at least 24 months after onset of treatment. Abbreviations: Dlco- diffusion capacity of the lungs for carbon monoxide.

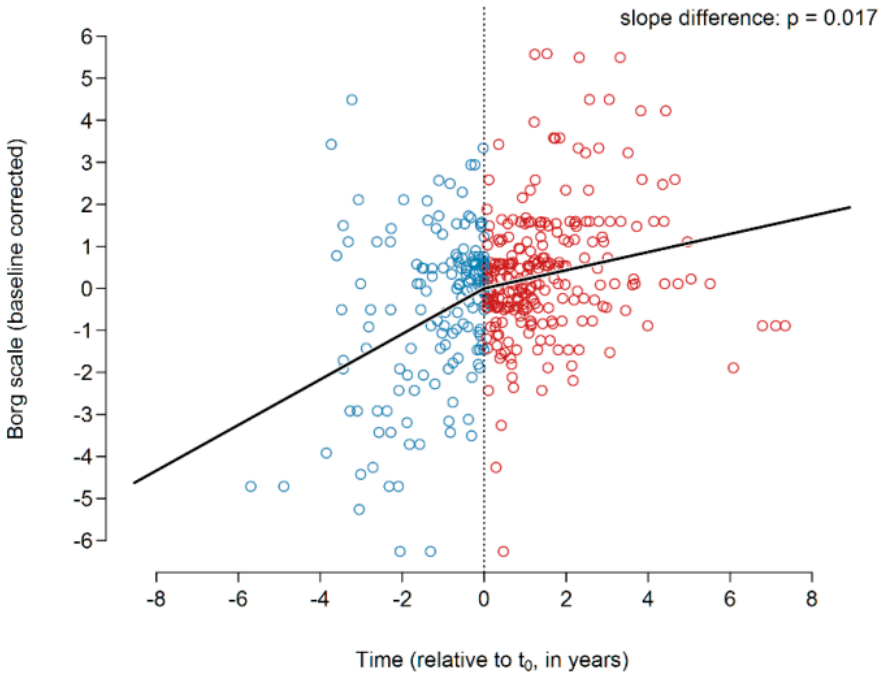

Suppl. Fig. 13. Borg dyspnea scale before and during pirfenidone treatment in patients surviving at least 24 months after onset of treatment.

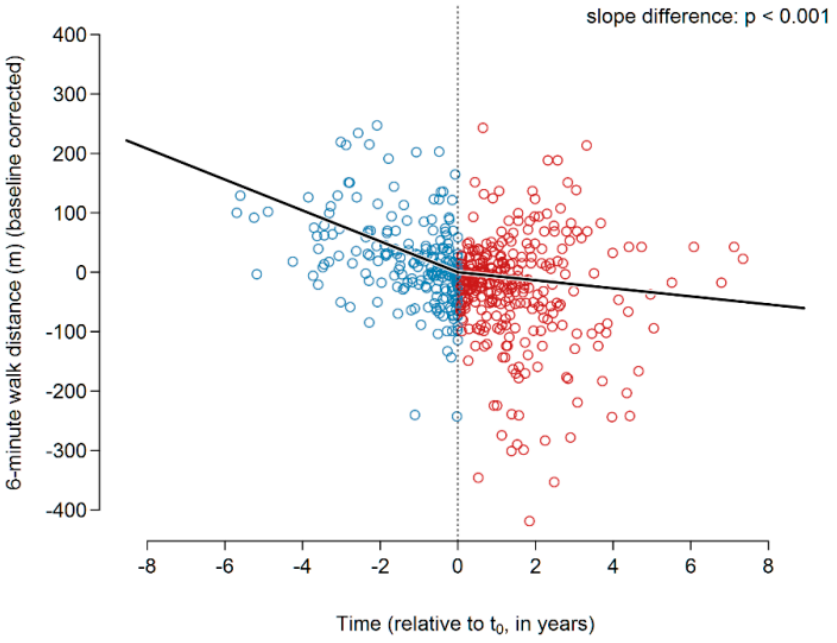

Suppl. Fig. 14. 6MWD before and during pirfenidone treatment in patients surviving at least 24 months after onset of treatment.

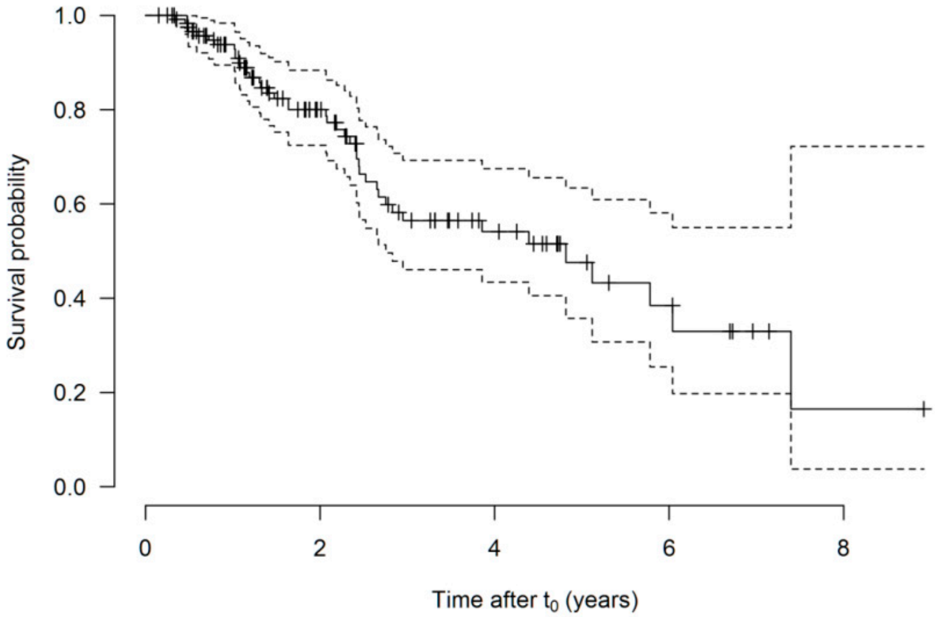

Suppl. Figure 15. Kaplan-Meier curve of patients under pirfenidone treatment. Given are estimate values and 95% confidence band.

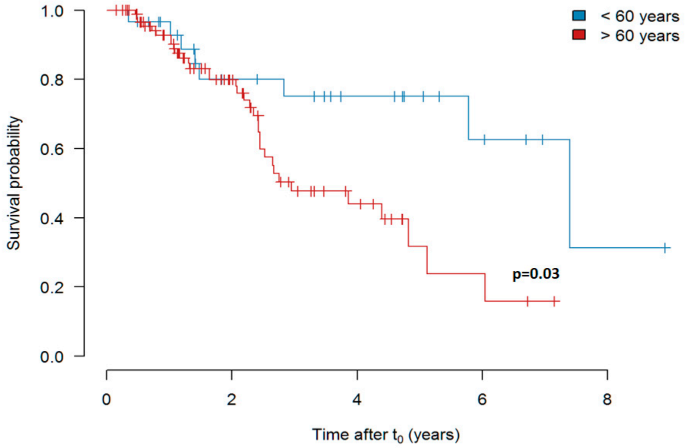

Suppl. Figure 16. Survival in dependency of the age at t0 (Kaplan-Meier curve shows estimate values).

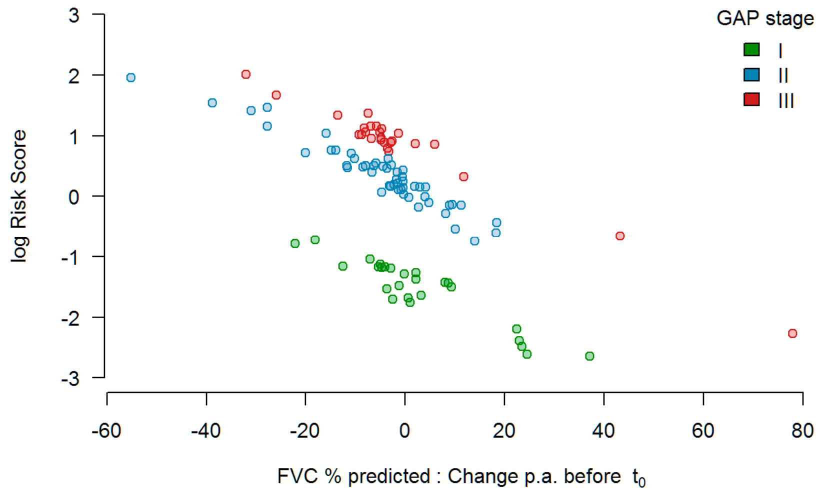

Suppl. Figure 17. Shown is scatter plot of the estimated risk (risk score with the highest number indicating the highest risk of death) depending on the FVC decline and GAP stage prior to initiation of treatment.

Supplementary Table 1. FVC decline per year under treatment with pirfenidone.

Year	Mean FVC decline per year (in liters)	Standard errors
-8	0.397	0.123
-7	0.183	0.071
-6	0.035	0.040
-5	-0.054	0.027
-4	-0.091	0.023
-3	-0.102	0.020
-2	-0.126	0.016
-1	-0.164	0.020
1	-0.068	0.015
2	-0.090	0.013
3	-0.096	0.017
4	-0.087	0.018

5	-0.061	0.021
6	-0.018	0.030
7	0.040	0.054
8	0.112	0.094

Presented are mean FVC values in liters and standard errors. Abbreviations: FVC- forced vital capacity.
